# Retraction: Assessing the Causal Association Between COVID-19 and Graves Disease: Mendelian Randomization Study

**DOI:** 10.2196/101185

**Published:** 2026-06-12

**Authors:** JMIR Publications Editorial Office

**Affiliations:** 1, JMIR Publications, 130 Queens Quay East, Suite 1100-1102Toronto, ON, Canada, 1 4165832040

The JMIR Publications Editorial Office is retracting the article “Assessing the Causal Association Between COVID-19 and Graves Disease: Mendelian Randomization Study” [1] due to concerns regarding potential manipulation of the submission process and authorship, and the integrity of the peer-review process.

Author Hua Yu responded, indicating that there was an undisclosed conflict of interest with one peer reviewer relating to a previous copublication but denied any undisclosed contributions to the manuscript. The Editorial Office has determined that this does not fully resolve the concerns regarding the submission process and authorship, and that there is sufficient evidence indicating that the peer-review process was compromised to proceed with a retraction.

All of the authors agree with the decision to retract the article.
